# Permutation-Based Block Code for Short Packet Communication Systems

**DOI:** 10.3390/s22145391

**Published:** 2022-07-19

**Authors:** Emil Faure, Anatoly Shcherba, Mykola Makhynko, Bohdan Stupka, Joanna Nikodem, Ruslan Shevchuk

**Affiliations:** 1 Faculty of Information Technology and Systems, Cherkasy State Technological University, 18006 Cherkasy, Ukraine; a.shcherba@chdtu.edu.ua (A.S.); b.a.stupka.fitis20@chdtu.edu.ua (B.S.); 2 State Scientific and Research Institute of Cybersecurity Technologies and Information Protection, 03142 Kyiv, Ukraine; 3 GoodLabs Studio Inc., Toronto, ON M5H 3E5, Canada; nmakhinko@goodlabs.studio; 4 Department of Computer Science and Automatics, University of Bielsko-Biala, 43-309 Bielsko-Biala, Poland; jnikodem@ath.bielsko.pl

**Keywords:** secure channel coding scheme, permutation, codeword, factorial code, block error-correcting code, permutation code, code size

## Abstract

This paper presents an approach to the construction of block error-correcting code for data transmission systems with short packets. The need for this is driven by the necessity of information interaction between objects of machine-type communication network with a dynamically changing structure and unique system of commands or alerts for each network object. The codewords of a code are permutations with a given minimum pairwise Hamming distance. The purpose of the study is to develop a statistical method for constructing a code, in contrast to known algebraic methods, and to investigate the code size. An algorithm for generating codewords has been developed. It can be implemented both by enumeration of the full set of permutations, and by enumeration of a given number of randomly selected permutations. We have experimentally determined the dependencies of the average and the maximum values of the code size on the size of a subset of permutations used for constructing the code. A technique for computing approximation quadratic polynomials for the determined code size dependencies has been developed. These polynomials and their corresponding curves estimate the size of a code generated from a subset of random permutations of such a size that a statistically significant experiment cannot be performed. The results of implementing the developed technique for constructing a code based on permutations of lengths 7 and 11 have been presented. The prediction relative error of the code size did not exceed the value of 0.72% for permutation length 11, code distance 9, random permutation subset size 50,000, and permutation statistical study range limited by 5040.

## 1. Introduction

The volume of information transfer including confidential information is continuously growing. According to the Statista Research Department report [1], over the next years up to 2025, global data creation is projected to grow to more than 180 zettabytes. A competitive environment has been created for designing and improving both attack systems and information security systems. These circumstances lead to an increase in the mathematical and logical complexity and degree of intellectualization of the used algorithms, processes, and technical means. As a result, the effectiveness and dependability [2] (reliability and security) of telecommunication systems and networks, as well as their components that implement data protection functions need to be improved.

Integrating methods of channel coding and cryptographic protection, or secure channel coding schemes, is one of the ways to increase the efficiency of information-processing tools, as well as to ensure data protection during its storage and transmission in telecommunication systems and networks.

Note that short packet transmission [3] is a key feature of modern wireless systems, ultra-reliable networks, sensor networks, massive machine-type communications (MTC), and IoT applications [4]. The prevalence of such systems and networks in the modern world requires the creation of new and the adaptation of existing approaches, to ensure the transmitted information integrity and confidentiality. In particular, the resources performance necessary for channel coding and cryptographic protection as well as the resources speed can play a decisive role.

This study considers an information interaction of MTC objects in a network with a dynamically changing structure. Each object of such a network, for example, a dynamic wireless sensor network [5], has its own unique system of commands or alerts. This system of commands or alerts forms an ensemble of messages to be agreed between the object of information interaction and other network participants.

### 1.1. Related Literature

Currently known secure-channel coding schemes are based on the McEliece cryptosystem [6,7,8,9], universal stochastic coding [10,11], ‘golden’ cryptography [12,13], perfect algebraic constructions [14,15], and the use of permutations [16,17].

This study develops an approach using permutations.

The methodology of integrated-information security based on non-separable factorial coding [18,19] uses a subset of the set of permutations π of numbers {0;1;…;M−1} as codewords. Each number {0;1;…;M−1} is encoded by a binary code with a fixed length of $l_{r}=\left\lceil \log_{2}M\right\rceil$ bits. Such information conversion allows getting a non-standard and redundant frame structure that does not require a separate field for the syncword, allows maintaining frame synchronization on the data signal, and allows the non-separable factorial code being used as a transport mechanism in short packet communications [20,21,22,23,24,25,26,27]. The cost of including syncwords is not negligible in such systems [28,29,30]. Using a non-separable factorial code makes it possible to effectively search for frame boundaries even with a bit error rate close to 0.5, which is important for information transmission under the conditions of strong noise [31,32]. In addition, non-separable factorial coding may be a suitable tool to implement a cross-layer integrated approach to security and achieve secure short-packet communication from the perspective of both cryptography and physical layer security [26,27].

Previous studies [33,34] investigate the ability of a non-separable factorial code to detect and correct communication channel errors. The efficiency of the code has been proven, which is achieved, among other factors, due to its synchronization properties [31,32]. The studies [33,34] use the binary Hamming distance between codewords.

In this paper, similarly to the error-correcting Reed-Solomon coding [35], we will consider symbols as elements of a codeword. This approach is of interest to ensure reliable transmission of permutations, in particular, for a three-pass cryptographic protocol based on permutations [36].

We introduce the following definition to distinguish between the binary Hamming distance used in previous studies [33,34] and the Hamming distance between permutations of symbols {0;1;…;M−1}.

**Definition** **1.** *The symbol Hamming distance*$D_{ij}$*between two permutations*$\pi_{i}$*and*$\pi_{j}$*is the number of symbol positions in which permutations*$\pi_{i}$*and*$\pi_{j}$*are different*.

It is obvious that $D_{ij}=D_{ji}$ and $0\leq D_{ij}\leq M$. In addition, $D_{ij}=0$ if and only if $\pi_{i}=\pi_{j}$.

**Definition** **2.** *A block code*$\left(M,D_{min}\right)$*is a code generated with a subset of permutations of length*M*with symbol Hamming distance*$D_{ij}\geq D_{min}$.

In this case, $D_{min}$ is the symbol code distance.

Let $N\left(M,D_{min}\right)$ be the $\left(M,D_{min}\right)$-code size equal to the number of its codewords.

Since the code size $N\left(M,D_{min}\right)$ determines the amount of information transmitted by each codeword equal to $\log_{2}\left(N\left(M,D_{min}\right)\right)$ bits, the use of a $\left(M,D_{min}\right)$-code of the maximum size is the most efficient in terms of channel capacity. The last statement also follows from the central problem of coding theory [37,38].

In the literature [39], the $\left(M,D_{min}\right)$-codes are called error correcting permutation codes. These codes are used for error correction of powerline communications using M-ary frequency shift keying modulation [40].

There are lower bounds for $N\left(M,D_{min}\right)$ (in particular, Gilbert–Varshamov bounds and their improvements) as well as algebraic techniques for constructing $\left(M,D_{min}\right)$-codes [39,41,42,43,44,45,46,47]. For example, N(M,2)=N(M,1)=M!, N(M,3)=M!/2, if M is a prime power then N(M,M−1)=M(M−1) and N(M+1,M−1)=(M+1)M(M−1) [41], N(11,8)=7920 and N(12,8)=95,040 [42]. Studies [39,43] use automorphism groups to provide $N\left(M,D_{min}\right)$ lower bounds. The authors of the literature [44] use permutations invariant under isometries. The study [45,46] uses sequential partition and extension, parallel partition and extension, and a modified Kronecker product operation. The recent study [47] improves $N\left(M,D_{min}\right)$ lower bounds using permutation rational functions.

In this study, in contrast to known algebraic methods, we present a statistical method for constructing a $\left(M,D_{min}\right)$-code and estimating its size $N\left(M,D_{min}\right)$. We also take into account the fact that the $\left(M,D_{min}\right)$-code must be unique for each object in the dynamic wireless sensor network, and the code agreement between the participants of the information exchange process can take place by applying a cryptographic protocol [36]. In such conditions, increasing the variability and unpredictability of the codeword ensemble is a necessary key condition for ensuring the protocol strength.

### 1.2. Main Contributions

We will generate codewords for a $\left(M,D_{min}\right)$-code by enumerating a set of permutations {π} of length M and selecting permutations with the symbol Hamming distance to all preselected permutations not exceeding the $D_{min}$ value. Constructing a $\left(M,D_{min}\right)$-code is complicated by the fact that when M increases, it is practically impossible to generate M! permutations.

The goal of the study is to determine the dependence of the code size $N\left(M,D_{min}\right)$ on the values of M and $D_{min}$ when using the proposed statistical method.

To achieve this goal, the following tasks must be solved.

- A statistical algorithm to generate codewords for a $\left(M,D_{min}\right)$-code must be developed and implemented.
- An analysis of the distribution frequency of a random value $N\left(M,D_{min}\right)$ for a given number of implementations of the codeword generating algorithm must be performed. The distribution law for $N\left(M,D_{min}\right)$ must be determined.
- The dependences of the average and the maximum $\left(M,D_{min}\right)$-code size, its standard deviation from the parameters M and $D_{min}$ must be explored.
- A technique to estimate a $\left(M,D_{min}\right)$-code size depending on parameters M and $D_{min}$ must be developed and applied.

### 1.3. Paper Structure

This paper is organized as follows: Section 2 describes an algorithm to generate a set of codewords, analyses the dependence of the $\left(M,D_{min}\right)$-code size on the values of M and $D_{min}$, and presents a technique for constructing an approximation polynomial for the code size dependencies; Section 3 shows the results of implementing the developed technique for M=11 and discusses the results, and Section 4 is the conclusion.

## 2. Materials and Methods

### 2.1. Algorithm to Generate Codewords

**Figure 1:**
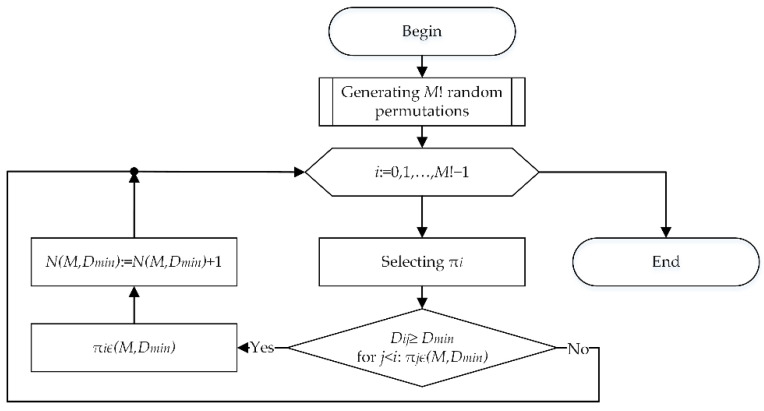
Algorithm to generate a set of codewords.

Figure 1 shows the algorithm to generate a set of codewords of a $\left(M,D_{min}\right)$-code.

Initially the set of codewords does not contain permutations. The initial complete set of M! permutations is generated randomly. The first permutation is selected and placed into the set of codewords being generated. Then the second permutation is selected and the Hamming distance to the first codeword is calculated for it. If the calculated distance is no less than the given $D_{min}$ value, the second permutation is also placed into the set of codewords. Otherwise, the next permutation from the initial set is selected. We continue the process of selecting permutations, calculating the Hamming distances to all selected codewords, and placing the permutation into the set of codewords if all calculated Hamming distances are no less than $D_{min}$ till all permutations of the initial set have been enumerated. After that, the number of permutations in the set of codewords is counted.

Constructing of a complete set of M! permutations can be implemented both by generating them in a certain, for example, lexicographic order with subsequent mixing, and by using random factorial numbers [0;M!−1] and their bijective transformation into permutations. At the same time, storing the permutation numbers (or the corresponding factorial numbers) instead of the permutations reduces the required amount of memory; however, due to additional transformations, it leads to an increase in the time to generate and output a permutation.

**Figure 2:**
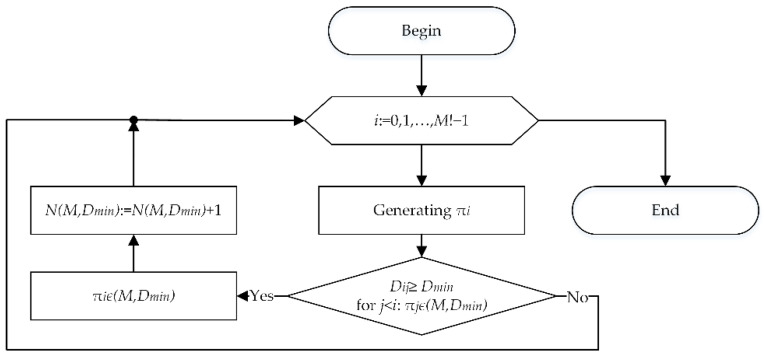
Algorithm to generate a set of codewords without storing the full set of permutations.

To reduce the amount of memory required to store the full set of M! permutations, the initial set of permutations can be generated simultaneously with their analysis. In this case, in the algorithm of Figure 1, there is no block for generating the initial set of permutations, and the block for selecting the next permutation is replaced by a block for generating the next permutation (Figure 2).

At the same time, the uniqueness check of the generated permutation is additionally implemented in the new block. Table 1 shows estimates of the mathematical expectation $\bar{N}\left(M,D_{min}\right)$ and the standard deviation $\sigma \left(N\left(M,D_{min}\right)\right)$ of the code size, as well as its maximum value $N_{max}\left(M,D_{min}\right)$ obtained as a result of implementing the algorithm shown in Figure 1 for 10,000 experiments with M=7 and $D_{min}=\left\{4,5,6\right\}$.

**Figure 3:**
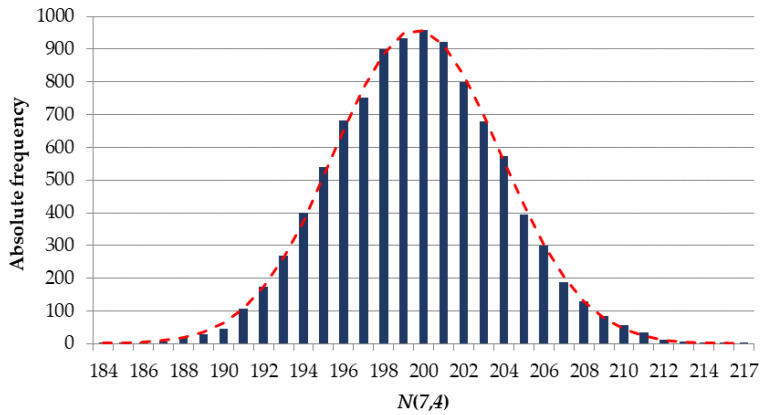
Histogram of the distribution of a random value N(7,4).

Figure 3 shows a histogram of the distribution of a random value $N\left(M,D_{min}\right)$ for M=7 and $D_{min}=4$.

Let the null hypothesis state that the distribution of a random value N(7,4) corresponds to a normal distribution. The use of Pearson’s chi-squared test $\chi^{2}$ [48] indicates that there is no reason to reject the null hypothesis with the achieved *p*-value (significance level) of 0.2768.

The normality of the distribution of a random value $N\left(M,D_{min}\right)$ is also confirmed for M=7 and $D_{min}=5$: p−value=0.6313. However, p−value=0.0000 for M=7 and $D_{min}=6$.

**Figure 4:**
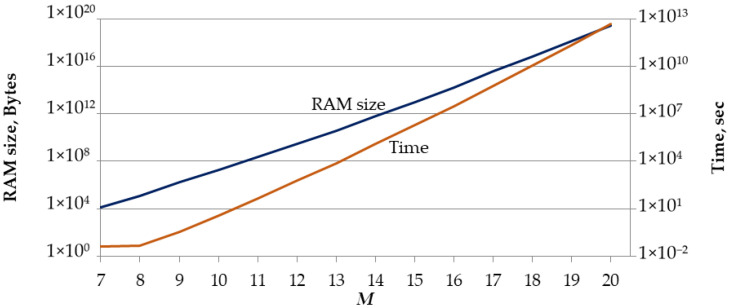
The required amount of memory and time to generate a complete set of M! permutations.

Note that as the value of M increases, the implementation of the algorithm shown in Figure 1 becomes more difficult, since the generation of a complete set of M! permutations requires a significant amount of memory and processor time (Figure 4). For example, storing of M!=39,916,800 (M=11) permutations using a fixed length binary code to encode permutation symbols requires 209.37 MB of memory; for M = 15 this amount is 8.92 TB. These calculations do not take into account the need to store service information. If we add service information then the memory amount required to form a complete set of permutations in the Python programming language [49] is 67 MB for M=9, 667 MB for M=10, 7.15 GB for M=11, and 70 GB for M=12. It is possible to somewhat reduce the amount of used memory by optimizing the program code. However, it is almost impossible to implement the algorithm shown in Figure 1 on a standard modern workstation when M≥12.

The average time to generate one permutation was determined experimentally by generating 1,000,000 permutations of a given length M.

All experiments in this research were implemented in the Python programming language [49] using the PyCharm Community Edition 2020.3 [50] integrated development environment on a desktop personal computer with the following parameters:

- OS—Windows 10
- CPU—Intel Core i5-10400F
- RAM 32Gb (2x16Gb dual channel 3200Mhz)
- GPU—GeForce GTX 1650 4Gb
- Hard Drive—SSD M.2 2280 1TB Samsung

Here, we provide the possibility to construct a $\left(M,D_{min}\right)$-code for the values of M that do not allow generating M! permutations in practice.

The approach proposed in this study is based on the following. The algorithm to generate a set of codewords shown in Figure 1 is preserved. At the same time, the initial set of permutations is a proper subset of the complete set of M! permutations. The size of such a proper subset is denoted by $N_{lim}$.

### 2.2. Algorithms to Generate the Initial Set of $N_{lim}$ Random Permutations

Permutations of the initial set will also be generated randomly. Here, we consider two algorithms:

1. Generating a random integer decimal number in the range [0;M!−1], converting the decimal number into a factorial number using division operations [51], and then converting the factorial number into a permutation [51];
2. Randomly generating individual digits of a factorial number and converting the factorial number into a permutation.

For example, permutation π=(3,2,4,0,1,5,6) with the basic permutation $\pi_{0}=\left(0,1,2,3,4,5,6\right)$ can be generated with both the first and the second algorithm. The first algorithm: A decimal number $A=2448_{\langle 10\rangle }$ is generated, converted into a factorial number $A=3220000_{\langle F\rangle }=3\cdot 6!+2\cdot 5!+2\cdot 4!+0\cdot 3!+0\cdot 2!+0\cdot 1!+0\cdot 0!$, and then converted into a permutation syndrome [52] $S_{F}=\left(3,2,2,0,0,0,0\right)$ and a permutation π=(3,2,4,0,1,5,6) itself. The second algorithm: Each element of the syndrome $S_{F}=\left(3,2,2,0,0,0,0\right)$ is generated separately and is then converted into a permutation π=(3,2,4,0,1,5,6).

**Figure 5:**
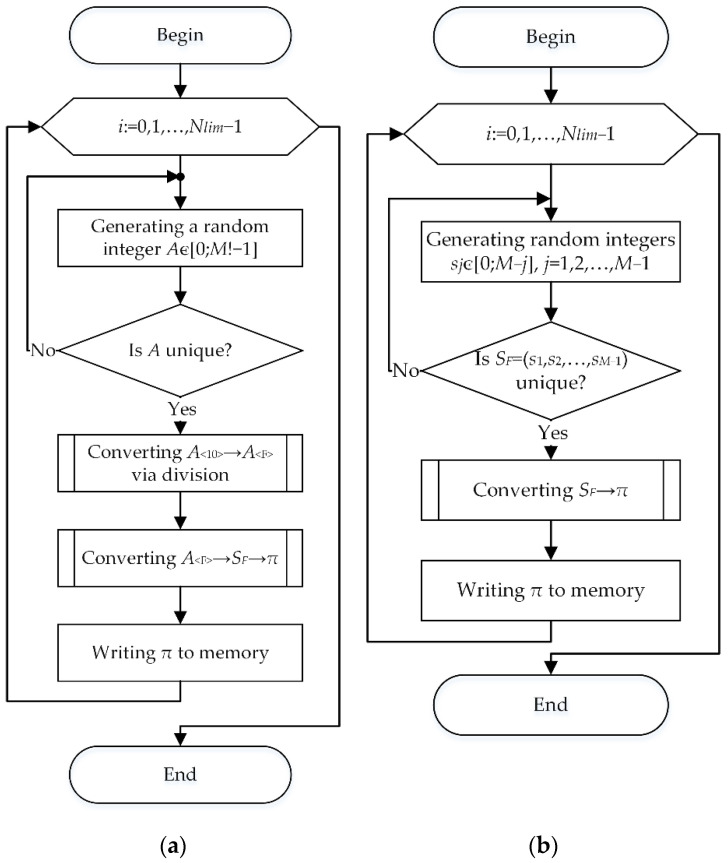
Algorithms to generate the initial set of $N_{lim}$ random permutations: (**a**) By division; (**b**) By generating digits.

Both of the above algorithms to generate the initial set of permutations control the uniqueness of permutations within the set (Figure 5).

Note that the above algorithms to generate $N_{lim}$ permutations can also be applied in the block for generating the next permutation of the algorithm in Figure 2. In this case, the algorithms will output the permutation for analysis instead of writing it to the memory.

**Figure 6:**
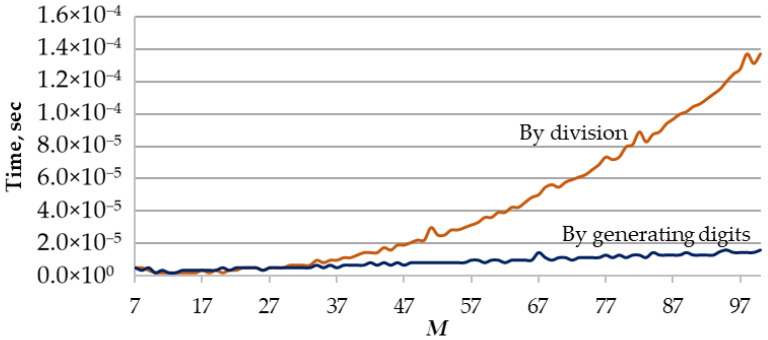
The average time to generate one factorial number using two different algorithms.

Comparing the speed of the two algorithms for generating random permutations shown in Figure 5, we evaluated the performance of only the distinctive parts of the presented algorithms, the procedures for generating factorial numbers. The average time to generate one factorial number (Figure 6) was calculated based on the results of the generation of 10,000 numbers.

The achieved graphs indicate that the time to generate a factorial number with the first algorithm (Figure 5) increases with an increase of the M value much faster than the second method. In addition, unlike the first algorithm, the processes for the second algorithm in Figure 5 are convenient for parallelization. This circumstance makes it possible to further increase the performance of the algorithm.

In this paper, we will use the second proposed algorithm to generate the initial set of $N_{lim}$ random permutations, $N_{lim}<M!$.

### 2.3. Dependence of the $\left(M,D_{min}\right)$-Code Size on the Values of M, $D_{min}$, and $N_{lim}$

We will use $\left(M,D_{min},N_{lim}\right)$ to denote block factorial code $\left(M,D_{min}\right)$ formed by a subset of $N_{lim}$ random permutations, and will use $N\left(M,D_{min},N_{lim}\right)$ to denote the size of $\left(M,D_{min},N_{lim}\right)$-code.

Next, we determine the dependence of the size $N\left(M,D_{min},N_{lim}\right)$ on the value of $N_{lim}$. Such dependence can be used both to determine the required value of $N_{lim}$ when designing a data transmission system with a $\left(M,D_{min}\right)$-code, and to evaluate the efficiency of the code constructed from $N_{lim}$ random permutations.

We will determine the dependence $N\left(M,D_{min},N_{lim}\right)$ experimentally. In this case, the $N_{lim}$ values are formed as follows.

Let $t_{0}=\ln\left(\ln M!\right)$. Then

(1) $$\ln\left(\ln N_{lim}\right)=t_{0}-\Delta \cdot t,$$

or

(2) $$N_{lim}=\exp\left(\exp\left(t_{0}-\Delta \cdot t\right)\right),$$

where Δ is a step; t=0,1,2,…,T.

Let M=7. Here, we accept Δ=0.04 for M=7. Values $N_{lim}$ for t=0,1,2,…,30 are given in Table 2.

**Figure 7:**
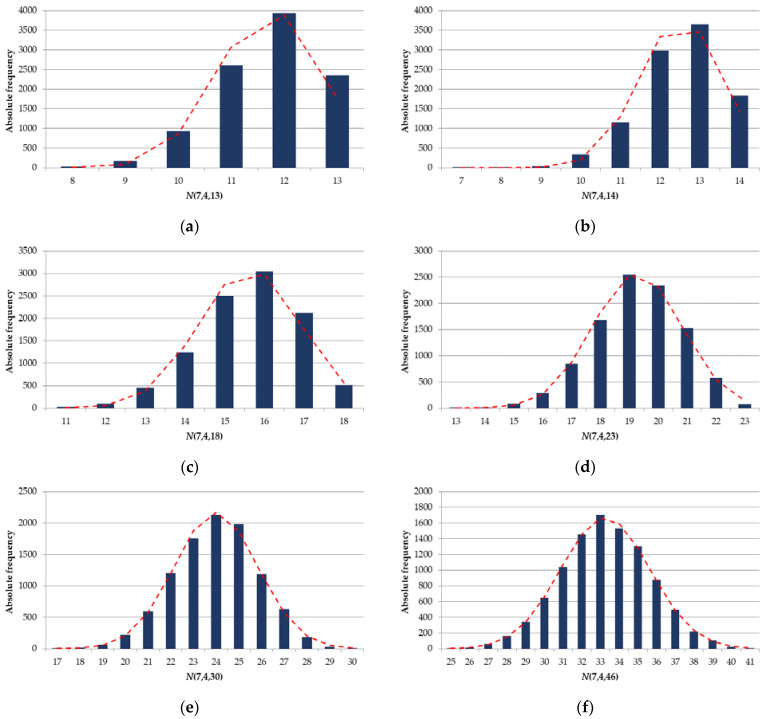
Histograms of the distribution of a random variable $N\left(7,4,N_{lim}\right)$ for: (**a**) $N_{lim}=13$; (**b**) $N_{lim}=14$; (**c**) $N_{lim}=18$; (**d**) $N_{lim}=23$; (**e**) $N_{lim}=30$; (**f**) $N_{lim}=46$.

Similarly to Figure 3, Figure 7 shows the histograms of the distribution of a random value $N\left(M,D_{min},N_{lim}\right)$ for $N_{lim}=\left\{13;14;18;23;30;46\right\}$ at M=7 and $D_{min}=4$, constructed as a result of 10,000 experiments.

By analogy with the distribution of a random value N(7,4), we accept the null statistical hypothesis, which states that a random variable $N\left(7,D_{min},N_{lim}\right)$ is normally distributed. We apply Pearson’s chi-square test to test the null hypothesis. Table 3 shows the *p-values* obtained for $N\left(7,D_{min},N_{lim}\right)$ at $D_{min}=\left\{4,5,6\right\}$ and $N_{lim}$ from Table 2.

In Table 3, the green highlights the cases where the normal distribution for $N\left(7,D_{min},N_{lim}\right)$ at the significance level of α=0.05 is confirmed; and the red highlights the cases where the normal distribution for $N\left(7,D_{min},N_{lim}\right)$ is not confirmed. These results can serve as evidence that at large $D_{min}$ values the normal distribution begins to be observed at smaller values of $N_{lim}$.

**Figure 8:**
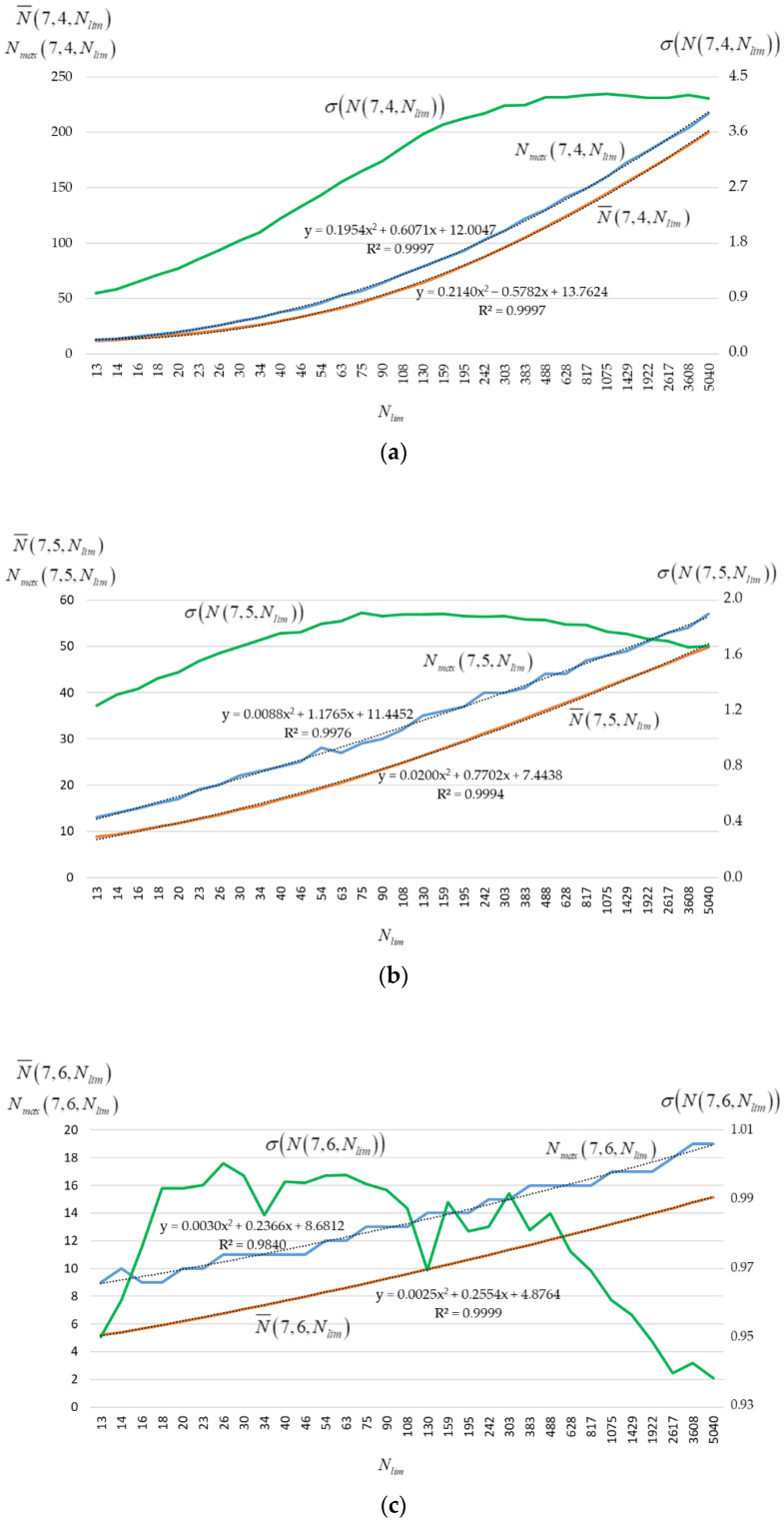
Graphs of $\bar{N}\left(M,D_{min},N_{lim}\right)$, $\sigma \left(N\left(M,D_{min},N_{lim}\right)\right)$ and $N_{max}\left(M,D_{min},N_{lim}\right)$ for M=7 and: (**a**) $D_{min}=4$; (**b**) $D_{min}=5$; (**c**) $D_{min}=6$.

Figure 8 shows the graphs of estimates of the mathematical expectation $\bar{N}\left(M,D_{min},N_{lim}\right)$, standard deviation $\sigma \left(N\left(M,D_{min},N_{lim}\right)\right)$, and the maximum value $N_{max}\left(M,D_{min},N_{lim}\right)$ of the $\left(M,D_{min}\right)$-code size against the value $N_{lim}$. The curves on Figure 8 are obtained as a result of 10,000 experiments for M=7 and $D_{min}=\left\{4,5,6\right\}$.

Figure 8 also shows the approximation curves [53] and equations, as well as the approximation reliability coefficient $R^{2}$ for the dependences of estimates of the mathematical expectation $\bar{N}\left(M,D_{min},N_{lim}\right)$ and the maximum value $N_{max}\left(M,D_{min},N_{lim}\right)$. The $R^{2}$ close to unity indicates an accurate description of the dependencies $\bar{N}\left(7,D_{min},N_{lim}\right)$ and $N_{max}\left(7,D_{min},N_{lim}\right)$ for $D_{min}=\left\{4,5,6\right\}$ by a second-degree polynomial of the form $y=ax^{2}+bx+c$. Table 4 summarizes the coefficients a, b, c for the $\bar{N}\left(7,D_{min},N_{lim}\right)$ and $N_{max}\left(7,D_{min},N_{lim}\right)$ approximation polynomials.

Note that x=T−t+1 according to Equation (2). Then the approximation functions can be easily calculated by setting the values of t for the required $N_{lim}$.

### 2.4. Technique for Constructing an Approximation Polynomial

To construct approximations for dependencies $\bar{N}\left(M,D_{min},N_{lim}\right)$ and $N_{max}\left(M,D_{min},N_{lim}\right)$ and, if necessary, to perform extrapolation to predict the behaviour of these functions at $N_{lim}$ values exceeding the upper limit of the range of their statistical study, it is necessary to perform the next steps:

1. To calculate $t_{0}=\ln\left(\ln M!\right)$, to set Δ and T values, and to calculate $t_{\min}=t_{0}-\Delta \cdot T$;
2. To generate dependencies $\bar{N}\left(M,D_{min},N_{lim}\right)$ and $N_{max}\left(M,D_{min},N_{lim}\right)$ for the range of $N_{lim}$ values determined in accordance with (2);
3. To determine approximation polynomials for $\bar{N}\left(M,D_{min},N_{lim}\right)$ and $N_{max}\left(M,D_{min},N_{lim}\right)$.

It is also possible to select the values of $N_{lim}$ for constructing dependencies $\bar{N}\left(M,D_{min},N_{lim}\right)$ and $N_{max}\left(M,D_{min},N_{lim}\right)$ in the opposite direction with respect to (1), from the smallest to the largest. In this case, the method to obtain approximations is as follows:

1. Values of $t_{\min}$, Δ, and T are chosen. Values of $N_{lim}$ are calculated using an expression
  (3) $$N_{lim}=\exp\left(\exp\left(t_{min}+\Delta \cdot t\right)\right),$$
  where t=0,1,2,…,T. It’s obvious that $T\leq \left(\ln\left(\ln M!\right)-t_{min}\right)/\Delta$;
2. Dependences $\bar{N}\left(M,D_{min},N_{lim}\right)$ and $N_{max}\left(M,D_{min},N_{lim}\right)$ are also formed for the range of $N_{lim}$ values determined in accordance with (3);
3. Quadratic approximation polynomials are calculated for $\bar{N}\left(M,D_{min},N_{lim}\right)$ and $N_{max}\left(M,D_{min},N_{lim}\right)$.

To obtain approximation polynomials of the form $y=at^{2}+bt+c$ in the obtained expressions of the form $y=ax^{2}+bx+c$, it is necessary to perform the replacement x=t+1.

## 3. Results

Here, we apply the developed method for M=11 when it is necessary to predict the average and the maximum number of codewords with $D_{min}=9$ formed by $N_{lim}=30,000$ and $N_{lim}=50,000$ random permutations.

**Figure 9:**
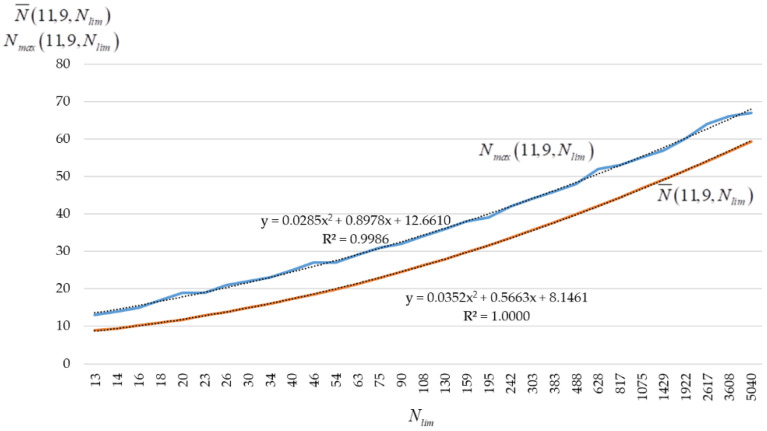
Graphs $\bar{N}\left(11,9,N_{lim}\right)$ and $N_{max}\left(11,9,N_{lim}\right)$ when Δ=0.04.

To construct approximations, we use the $N_{lim}$ values given in Table 2 for the step Δ=0.04 at $t_{0}=\ln\left(\ln7!\right)=2.1430$. Figure 9 shows the graphs of the estimates of the mathematical expectation $\bar{N}\left(11,9,N_{lim}\right)$ and the maximum value $N_{max}\left(11,9,N_{lim}\right)$ of the code size $\left(11,9,N_{lim}\right)$ against the value $N_{lim}=\left\{13;16;20;\ldots ;2617;5040\right\}$. Each value of $\bar{N}\left(11,9,N_{lim}\right)$ and $N_{max}\left(11,9,N_{lim}\right)$ was formed as a result of 10,000 experiments.

If we place values $N_{lim}=30,000$ and $N_{lim}=50,000$ into the expression (1) $\ln\left(\ln N_{lim}\right)=t_{0}-\Delta \cdot t$, and calculate the corresponding values of $t=\frac{1}{0.04}\ln\frac{\ln5040}{\ln N_{lim}}$, we can get $t\left(N_{lim}=30,000\right)=-4.7498$, $t\left(N_{lim}=50,000\right)=-5.9588$. Note that x=T−t+1, T=30. Then the predicted values $\bar{N}\left(11,9,N_{lim}\right)$ and $N_{max}\left(11,9,N_{lim}\right)$ are equal to 73.3836 and 81.2250 for $N_{lim}=30,000$ and 77.1631 and 84.8191 for $N_{lim}=50,000$.

**Figure 10:**
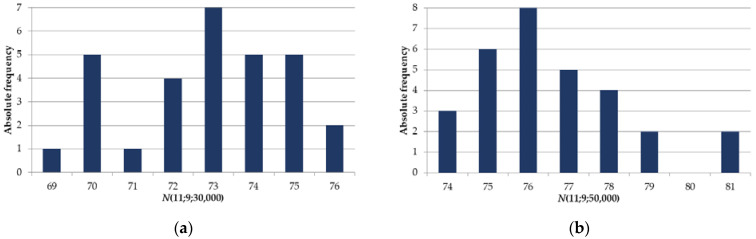
Histograms of the distribution of a random value $N\left(11,9,N_{lim}\right)$ for: (**a**) $N_{lim}=30,000$; (**b**) $N_{lim}=50,000$.

Figure 10 shows the histograms of the distribution of random values $N\left(11,9,3\times 10^{4}\right)$ and $N\left(11,9,5\times 10^{4}\right)$ constructed as a result of K=30 experiments. The resulting average values are 72.8667 and 76.5667 (maximum values are 76 and 81).

Here, we determine the confidence interval for the obtained sample means [54]:

$$\bar{N_{sample}}\left(M,D_{min},N_{lim}\right)-\frac{s}{\sqrt{K}}t_{\alpha ,K-1}<\bar{N}\left(M,D_{min},N_{lim}\right)<\bar{N_{sample}}\left(M,D_{min},N_{lim}\right)+\frac{s}{\sqrt{K}}t_{\alpha ,K-1},$$

where $\bar{N_{sample}}\left(M,D_{min},N_{lim}\right)$ is the sample mean;

$s=\sqrt{\frac{K}{K-1}}\cdot \sigma_{sample}$ is the corrected sample standard deviation, $\sigma_{sample}$ is the sample standard deviation;

K is the number of experiments (K=30);

$t_{\alpha /2,K-1}$ is the the upper α/2 quantile of Student’s *t*-distribution with K−1 degrees of freedom.

Let α=0.05. Then, the confidence interval is (72.1346;73.5987) for $\bar{N}\left(11.9,3\times 10^{4}\right)$, and it is (75.8825;77.2509) for $\bar{N}\left(11.9,5\times 10^{4}\right)$.

The predicted values of 73.3836 and 77.1631 fall within the indicated confidence intervals.

**Figure 11:**
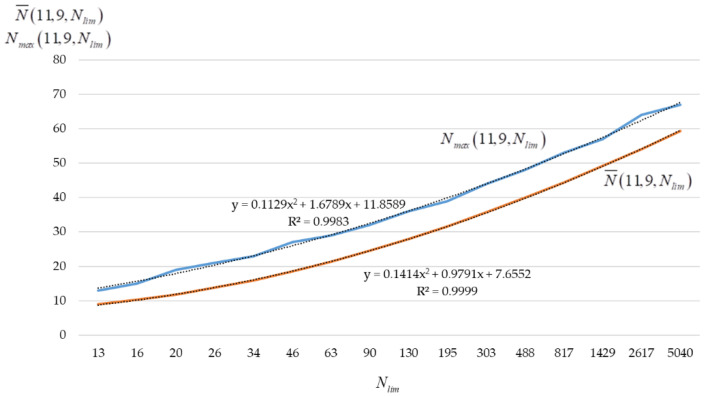
Graphs $\bar{N}\left(11,9,N_{lim}\right)$ and $N_{max}\left(11,9,N_{lim}\right)$ when Δ=0.08.

Then, let Δ=0.08 when $t_{0}=\ln\left(\ln7!\right)=2.1430$. Figure 11 shows the graphs of the estimates of the mathematical expectation $\bar{N}\left(11,9,N_{lim}\right)$ and the maximum value $N_{max}\left(11,9,N_{lim}\right)$ of the $\left(11,9,N_{lim}\right)$-code size against the value $N_{lim}=\left\{13;16;20;\ldots ;2617;5040\right\}$. Each value of $\bar{N}\left(11,9,N_{lim}\right)$ and $N_{max}\left(11,9,N_{lim}\right)$ was formed as a result of 10,000 experiments.

By placing values $N_{lim}=30,000$ and $N_{lim}=50,000$ into the expression (1) $\ln\left(\ln N_{lim}\right)=t_{0}-\Delta \cdot t$, and calculating the corresponding values of $t=\frac{1}{0.08}\ln\frac{\ln5040}{\ln N_{lim}}$ we get $t\left(N_{lim}=30,000\right)=-2.3749$, $t\left(N_{lim}=50,000\right)=-2.9794$. Taking into account that x=T−t+1, T=15, the predicted values $\bar{N}\left(11,9,N_{lim}\right)$ and $N_{max}\left(11,9,N_{lim}\right)$ are equal to 73.3922 and 80.8342 for $N_{lim}=30,000$ and 77.1775 and 84.3992 for $N_{lim}=50,000$.

Let the step Δ be further increased. Table 5 summarizes the predicted values $\bar{N}\left(11,9,N_{lim}\right)$ and $N_{max}\left(11,9,N_{lim}\right)$ for $N_{lim}=30,000$ and $N_{lim}=50,000$ when Δ={0.04;0.08;0.012;…;0.4;0.44}.

Table 5 shows that all predicted values fall within the indicated confidence intervals (72.1346;73.5987) for $\bar{N}\left(11.9,3\times 10^{4}\right)$ and (75.8825;77.2509) for $\bar{N}\left(11.9,5\times 10^{4}\right)$.

Here, we calculate and present in Table 6 the relative prediction error for the values given in Table 5. We assume that the maximum number of reference points (T=30) forms the most accurate prediction.

The results in Table 6 indicate that three points as far as possible from each other (T=2) are sufficient to obtain an approximation curve (an approximation polynomial of the second degree). At the same time, the authors recommend using four points (T=3) to construct such a curve.

Here, we discuss the study results.

The proposed algorithm to generate codewords allows for the provision of the necessary technical result of constructing $\left(M,D_{min}\right)$-code with the required code distance. However, the obtained $N\left(M,D_{min}\right)$ values do not reach the known lower bounds [39,41,42,43,44,45,46,47]. For example, $N_{max}\left(11,9,5040\right)=67$. At the same time, paper [39] gives the lower bound of 154 for the (11,9)-code size. The corresponding values for M=7 are $N_{max}\left(7,6,5040\right)=19$ vs. the lower bound of 42 and $N_{max}\left(7,5,5040\right)=57$ vs. the lower bound of 77 in [39]. However, we cannot say that the result is negative. First, in this study, we used not an algebraic, but a statistical method for code construction. Second, the proposed statistical method, unlike the algebraic method, allows for the construction of a unique system of commands or alerts for dynamic wireless sensor network objects. Note also that increasing the $N\left(M,D_{min}\right)$-code size may lead to a decrease in the number of different possible $\left(M,D_{min}\right)$-codes, which can be constructed for the defined values of M, $D_{min}$, and $N_{lim}$. In turn, the number of different possible $\left(M,D_{min}\right)$-codes is important for applying the $\left(M,D_{min}\right)$-code both in secure-channel coding schemes and for constructing a unique system of commands or alerts for MTC objects. At the same time, we do not deny the need to continue the search for new effective and fast statistical methods for $\left(M,D_{min}\right)$-code construction or to improve the proposed method. Determining the balance between the $\left(M,D_{min}\right)$-code size and the number of possible different $\left(M,D_{min}\right)$-codes is an actual problem that can be the subject for further research.

The study has shown that the relative error in predicting the size of $\left(M,D_{min}\right)$-code increases with increasing the hypothetical number $N_{lim}$ of permutations in the initial set, as well as with increasing the step Δ. However, the nature of this dependence is not obvious and can be further investigated.

## 4. Conclusions

In this paper, we have developed and implemented a statistical algorithm to generate codewords of a $\left(M,D_{min}\right)$-code by enumerating a set of permutations {π} of length M and selecting permutations with the symbol Hamming distance to all preselected codewords not exceeding the $D_{min}$ value.

We applied two algorithms to generate a random factorial number. The first algorithm is based on the conversion from a random decimal number by division, and the second algorithm is based on the random generation of individual digits of a factorial number. We found that the second method is faster.

We have determined experimentally the dependences of the average and the maximum values of the size $N\left(M,D_{min},N_{lim}\right)$ of a $\left(M,D_{min}\right)$-code constructed from a subset of $N_{lim}$ permutations, on the value of $N_{lim}$.

A technique to compute approximation quadratic polynomials for the determined dependences of the average and the maximum values of the $\left(M,D_{min}\right)$-code size has been developed. A key feature of this technique is to use the function (1) of a double logarithm $\ln\left(\ln N_{lim}\right)$ and to use a quadratic polynomial. The approximation polynomials and their corresponding curves can be used to extrapolate the dependencies and predict their behavior at $N_{lim}$ values exceeding the upper limit of their statistical study range.

Finally, we confirmed the effectiveness of the developed technique to estimate the average and the maximum size values $N\left(11.9,N_{lim}\right)$ for $N_{lim}=30,000$ and $N_{lim}=50,000$ at the upper limit of the statistical study range $N_{lim}=5040$. The prediction relative error of $\left(M,D_{min}\right)$-code size did not exceed the value of 0.72% obtained for $N_{lim}=50,000$ and Δ=0.44.

## Figures and Tables

**Table 1:** Values of $\bar{N}\left(M,D_{min}\right)$, $\sigma \left(N\left(M,D_{min}\right)\right)$, and $N_{max}\left(M,D_{min}\right)$ for M=7 and $D_{min}=\left\{4,5,6\right\}$.

$D_{min}$	4	5	6
$\bar{N}\left(7,D_{min}\right)$	199.6787	49.8305	15.1698
$\sigma \left(N\left(7,D_{min}\right)\right)$	4.1532	1.6693	0.9384
$N_{max}\left(M,D_{min}\right)$	217	57	19

**Table 2:** Dependence of $N_{lim}$ for M=7 and Δ=0.04 when t=0,1,2,…,30.

t	$N_{lim}$	t	$N_{lim}$	t	$N_{lim}$	t	$N_{lim}$	t	$N_{lim}$	t	$N_{lim}$
0	5040	6	817	12	195	18	63	24	26	30	13
1	3608	7	628	13	159	19	54	25	23		
2	2617	8	488	14	130	20	46	26	20		
3	1922	9	383	15	108	21	40	27	18		
4	1429	10	303	16	90	22	34	28	16		
5	1075	11	242	17	75	23	30	29	74		

**Table 3:** *p*-values for $N\left(7,D_{min},N_{lim}\right)$.

$N_{lim}\;\backslash \;D_{min}$	4	5	6
13	0.000000	0.193433	0.000002
14	0.000000	0.367169	0.000000
16	0.000000	0.380055	0.000090
18	0.000000	0.589350	0.000028
20	0.000000	0.288059	0.000000
23	0.000000	0.755697	0.000000
26	0.000000	0.000328	0.337444
30	0.000012	0.000049	0.000011
34	0.299914	0.000002	0.013646
40	0.035342	0.002416	0.000689
46	0.421327	0.008021	0.000055
54	0.622645	0.000000	0.000001
63	0.755772	0.000324	0.000297
75	0.832253	0.000087	0.000013
90	0.104076	0.713204	0.018065
108	0.653265	0.647142	0.001913
130	0.978050	0.010121	0.000001
159	0.076289	0.000081	0.000449
195	0.061514	0.003801	0.000813
242	0.427242	0.066269	0.026604
303	0.489814	0.410349	0.025393
383	0.044070	0.053179	0.020188
488	0.527648	0.076959	0.031807
628	0.000791	0.594741	0.336770
817	0.242391	0.926065	0.037511
1075	0.949684	0.470563	0.932577
1429	0.019384	0.038698	0.093501
1922	0.912574	0.428931	0.177253
2617	0.085210	0.085414	0.000893
3608	0.545212	0.143008	0.000000
5040	0.276788	0.631289	0.000000

**Table 4:** Coefficients of quadratic approximation polynomials for $\bar{N}\left(7,D_{min},N_{lim}\right)$ and $N_{max}\left(7,D_{min},N_{lim}\right)$.

	$\bar{N}\left(7,D_{min},N_{lim}\right)$			$N_{max}\left(7,D_{min},N_{lim}\right)$		
$D_{min}$	a	b	c	a	b	c
4	0.2140	−0.5782	13.7624	0.1954	0.6071	12.0047
5	0.0200	0.7702	7.4438	0.0088	1.1765	11.4452
6	0.0025	0.2554	4.8764	0.0030	0.2366	8.6812

**Table 5:** Predicted values $\bar{N}\left(11,9,N_{lim}\right)$ and $N_{max}\left(11,9,N_{lim}\right)$ for $N_{lim}=30,000$ and $N_{lim}=50,000$ depending on the step Δ value.

Δ	T	Expected $\bar{N}\left(11,9,N_{lim}\right)\;$		Expected $N_{max}\left(11,9,N_{lim}\right)\;$	
		$N_{lim}=30,000\;$	$N_{lim}=50,000\;$	$N_{lim}=30,000\;$	$N_{lim}=50,000\;$
0.04	30	73.3836	77.1631	81.2250	84.8191
0.08	15	73.3922	77.1775	80.8342	84.3992
0.12	10	73.3180	77.0940	80.1974	83.6976
0.16	7	73.2486	77.0101	79.6796	83.1427
0.20	6	73.2849	77.0551	79.4527	82.8157
0.24	5	73.2955	77.0786	79.9968	83.4801
0.28	4	73.1316	76.8810	79.6330	82.9191
0.32	3	72.9731	76.6821	80.5106	84.1495
0.36	3	73.1561	76.9123	80.0525	83.5624
0.4	3	73.1844	76.9536	79.0658	82.3375
0.44	2	72.9079	76.6054	79.6497	83.0483

**Table 6:** Relative prediction error, %.

Δ	T	Expected $\bar{N}\left(11,9,N_{lim}\right)$	
		$N_{lim}=30,000$	$N_{lim}=50,000$
0.04	30	0.00	0.00
0.08	15	0.01	0.02
0.12	10	0.09	0.09
0.16	7	0.18	0.20
0.20	6	0.13	0.14
0.24	5	0.12	0.11
0.28	4	0.34	0.37
0.32	3	0.56	0.62
0.36	3	0.31	0.33
0.4	3	0.27	0.27
0.44	2	0.65	0.72

## Data Availability

All initial data, program codes will be provided upon request to the correspondent’s e-mail with appropriate justification.
